# Supplementary figures and images for: Velocities of hippocampal traveling waves are proportional to their coherence frequency
Source: PLoS One. 2025 Feb 21;20(2):e0313900. doi: 10.1371/journal.pone.0313900 (PMC11844891; doi:10.1371/journal.pone.0313900)

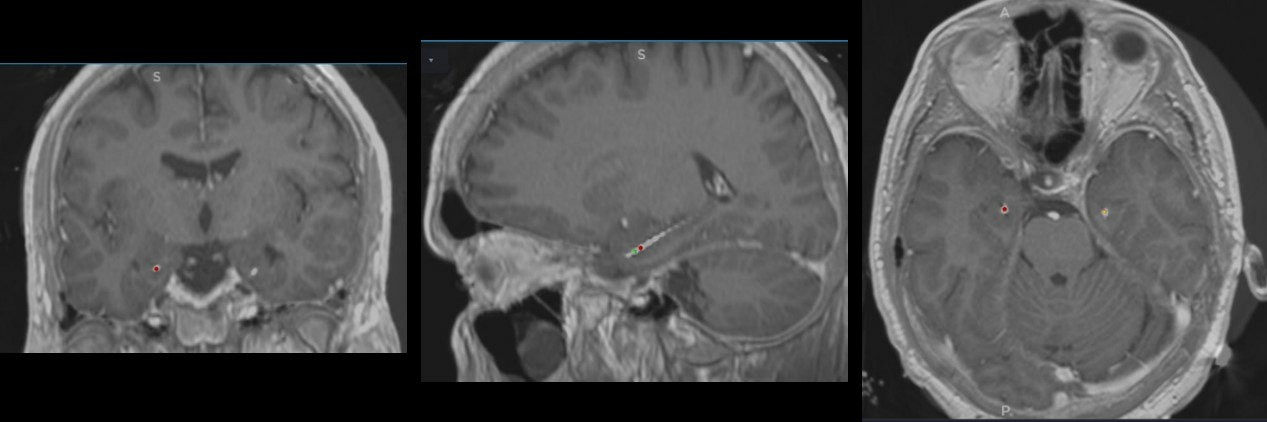

Supplement: S1 Fig — The red dot indicates contact number 5 on the SEEG electrode inserted to the left hippocampus. (TIF) [file pone.0313900.s001.tif]

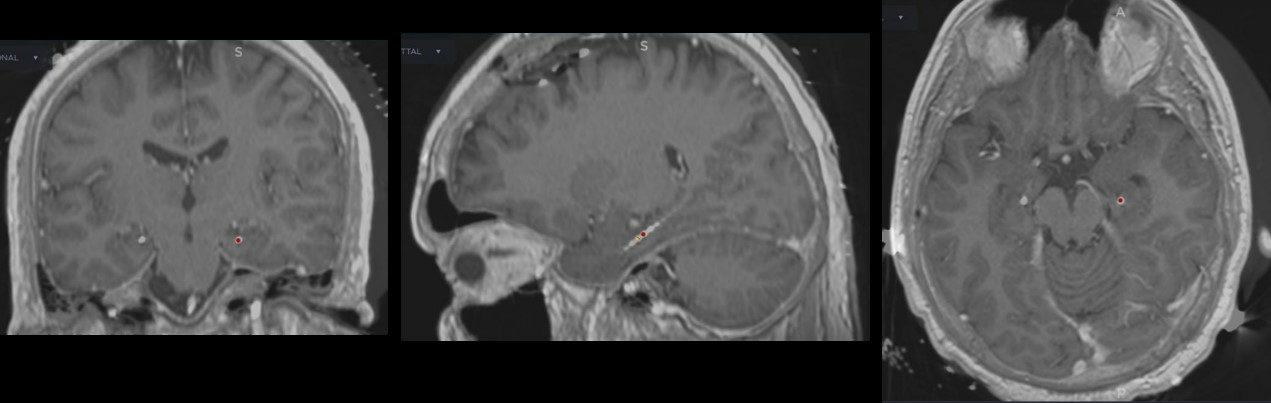

Supplement: S2 Fig — The red dot indicates contact number 5 on the SEEG electrode inserted to the right hippocampus. (TIF) [file pone.0313900.s002.tif]

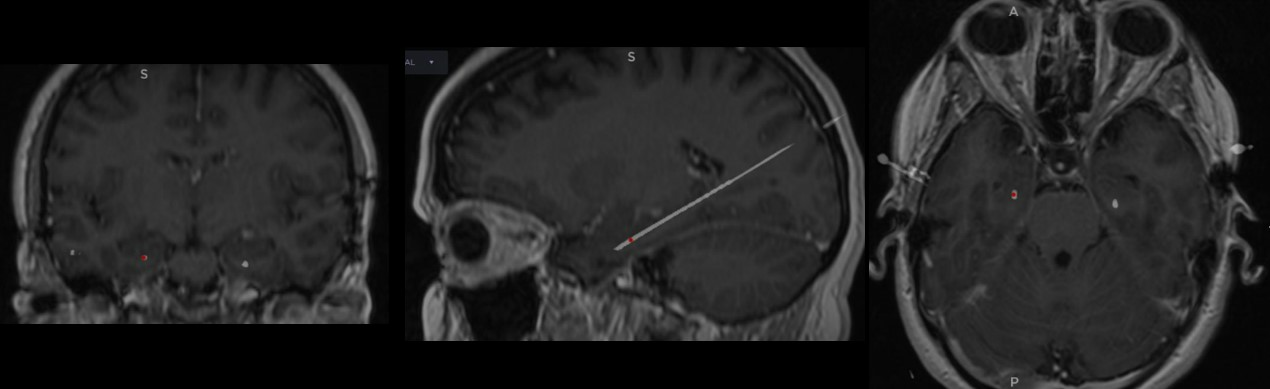

Supplement: S3 Fig — The red dot indicates contact number 4 on the SEEG electrode inserted to the left hippocampus. (TIF) [file pone.0313900.s003.tif]

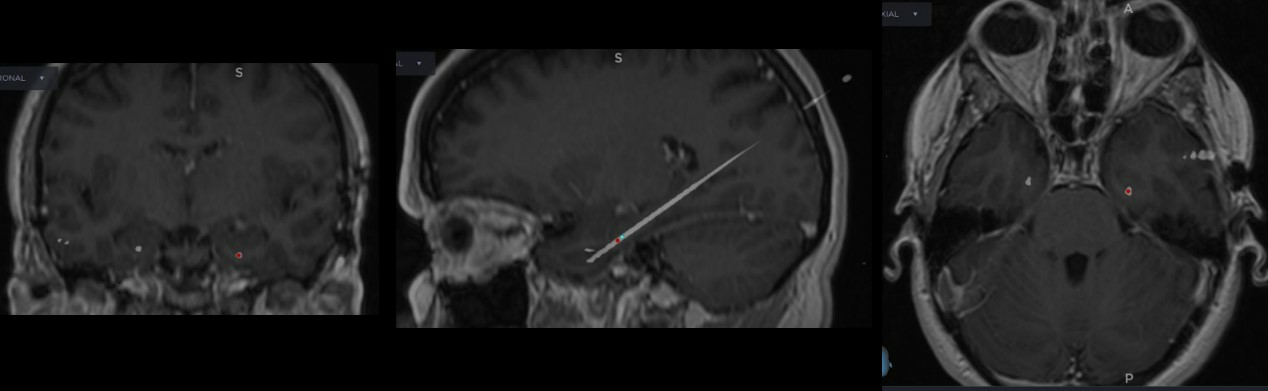

Supplement: S4 Fig — The red dot indicates contact number 5 on the SEEG electrode inserted to the right hippocampus. (TIF) [file pone.0313900.s004.tif]

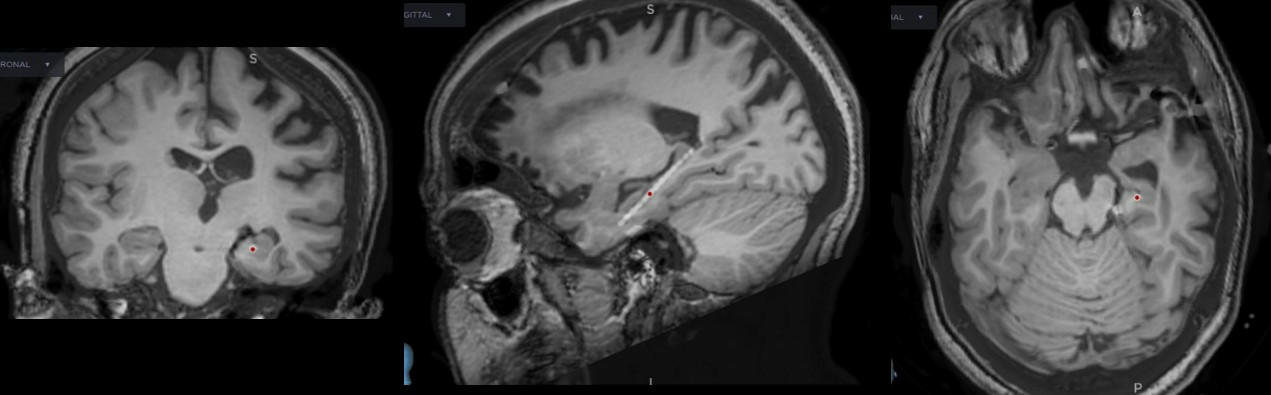

Supplement: S5 Fig — The red dot indicates contact number 6 on the SEEG electrode inserted to the right hippocampus. (TIF) [file pone.0313900.s005.tif]

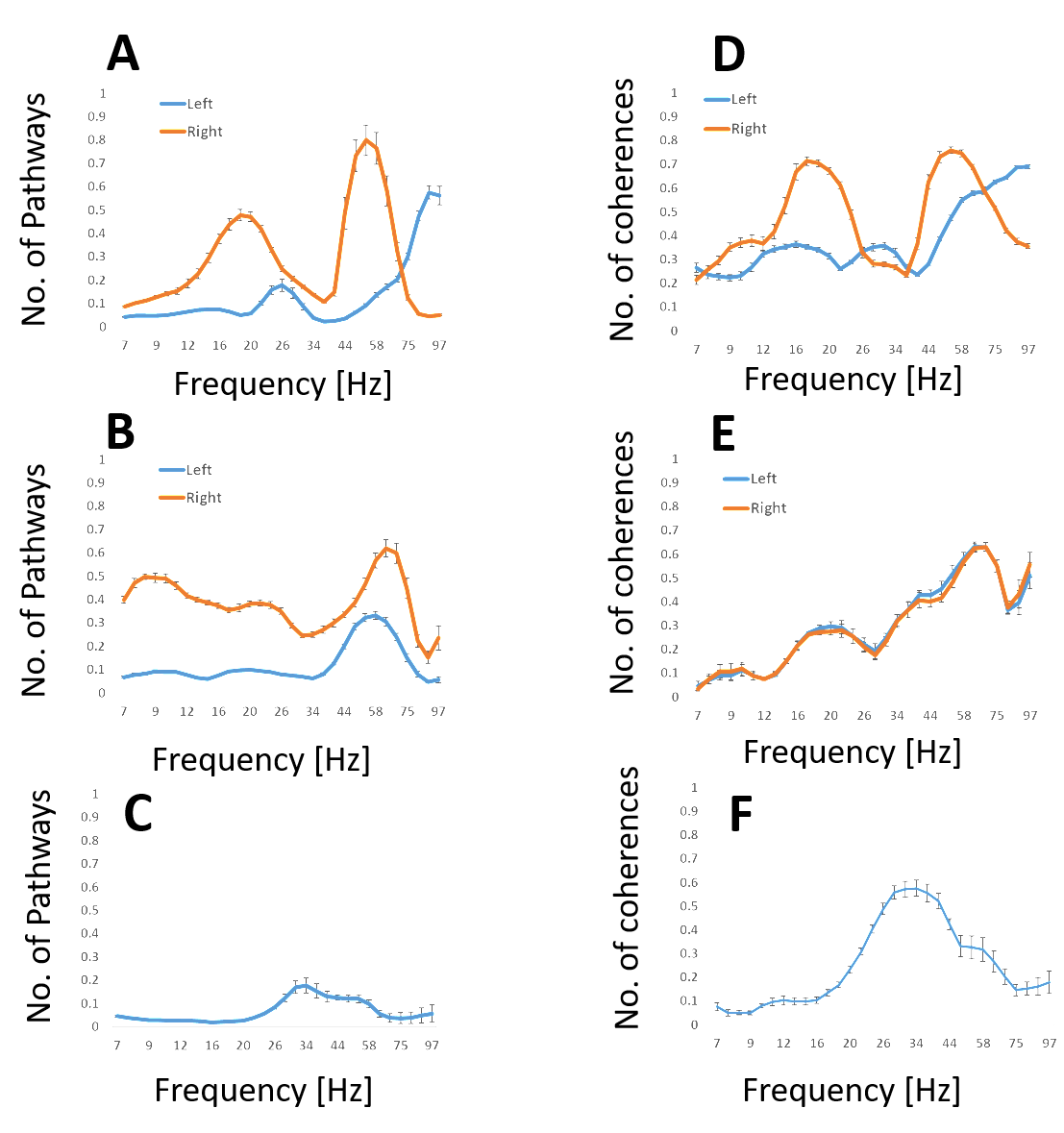

Supplement: S6 Fig — A and D represent the left and right hippocampi of subject 1, B and E represent the left and right hippocampi of subject 2, and C and F represent the right hippocampus of subject 3. Normalization was to the maximum number of pathways/pairs that is the number of combinations with all contact’ signals. (TIF) [file pone.0313900.s006.tif]
