# Supplementary material for: Velocities of hippocampal traveling waves are proportional to their coherence frequency
Source: PLoS One. 2025 Feb 21;20(2):e0313900. doi: 10.1371/journal.pone.0313900 (PMC11844891; doi:10.1371/journal.pone.0313900)
Supplement: S1 Table — E1 to E4 are the four contact signals. (DOCX) [file pone.0313900.s007.docx]

**Table 1**

| **PW number** | **Pathway** |
| --- | --- |
| 1 | E1-E2-E3-E4 |
| 2 | E2-E3-E4-E1 |
| 3 | E3-E4-E1-E2 |
| 4 | E4-E1-E2-E3 |
| 5 | E1-E2-E4-E3 |
| 6 | E2-E4-E3-E1 |
| 7 | E3-E1-E2-E4 |
| 8 | E4-E3-E1-E2 |
| 9 | E1-E3-E4-E2 |
| 10 | E3-E4-E2-E1 |
| 11 | E4-E2-E1-E3 |
| 12 | E2-E1-E3-E4 |
| 13 | E1-E4-E3-E2 |
| 14 | E2-E1-E4-E3 |
| 15 | E3-E2-E1-E4 |
| 16 | E4-E3-E2-E1 |
| 17 | E1-E3-E2-E4 |
| 18 | E2-E4-E1-E3 |
| 19 | E3-E2-E4-E1 |
| 20 | E4-E1-E3-E2 |
| 21 | E1-E4-E2-E3 |
| 22 | E2-E3-E1-E4 |
| 23 | E3-E1-E4-E2 |
| 24 | E4-E2-E3-E1 |

**Table 1:** A list of all four-node pathways. E1 to E4 are the four contact signals.
