# Supplementary material for: Velocities of hippocampal traveling waves are proportional to their coherence frequency
Source: PLoS One. 2025 Feb 21;20(2):e0313900. doi: 10.1371/journal.pone.0313900 (PMC11844891; doi:10.1371/journal.pone.0313900)
Supplement: Attachment — Submitted filename: Letter to referee.docx [file pone.0313900.s008.docx]

We thank the editor and the reviewer for their valuable critiques and comments. Below, we provide our responses and describe the changes made in accordance with these suggestions:

**Editor Comments**

1. **Please include a caption for figures 1, 2, 3, 4, 5, and 6.**
   Captions for these figures have been added at the end of the manuscript.
2. **Please include a caption for table 1.**
   A caption for table 1 has been added in the revised version. Thank you.
3. **Restriction on data sharing:**
   The local field potential (LFP) data used in this manuscript was obtained from human hippocampal recordings of epileptic patients. Due to ethical, legal, and regulatory restrictions, we have specified data-sharing availability as "Available upon reasonable request." The restrictions are as follows:
   1. **Informed Consent**: Patients provided explicit informed consent for their data to be used exclusively by the Hadassah research team and not by other researchers.
   2. **Data Use Agreements (DUA)**: Any researcher receiving this data must sign a DUA outlining permitted uses and explicitly prohibiting unauthorized distribution or commercial use.
   3. **Research Only**: The data is strictly limited to non-commercial research purposes within specific fields, such as epilepsy research.
   4. **Ethics Committee Approval**: Any research involving LFP data sharing or secondary use requires prior approval from our ethics committee.
   5. **Sensitive Information**: The LFP data contains details about seizure events, which makes it particularly sensitive. Consequently, restrictions limit access to seizure-related data to authorized personnel.
   6. **Potential for Re-identification**: Epileptic patterns and associated clinical metadata may increase the risk of patient re-identification, necessitating additional safeguards.
4. **Please include your tables as part of your main manuscript and remove the individual files.**
   This has been completed. Thank you.
5. **Please include captions for your Supporting Information files at the end of your manuscript.**
   Captions for the Supporting Information files have been added at the end of the manuscript. Thank you.

**Reviewer Comments**

1. This is a very interesting paper proposing a new method based on 4-node recording configurations, to detect multiple traveling waves from electrophysiological recordings. The paper is certainly worth publishing but a couple of point should be clarified.

Thank you.

2. First, it is not clear how the method compares with previous methods, in particular based on Hilbert transforms. The study of Muller et al. Nature Communications 2014 was the first to reveal traveling waves in monkey (as far as I am aware), and were demonstrated there with voltage-sensitive dye (VSD) imaging. In that study they used Hilbert transforms to detect traveling waves from the data and represent the data with phase maps, where traveling waves appear more naturally. (BTW they had an instantaneous frequeny around 10Hz, and velocity about 0.5 ms, which I think matches your estimates). What do you get if you calculate the phase map with the 4 nodes? Should you not get the same information? Please clarify how your method differs from using Hilbert phase, or how they are related.

We thank the reviewer for referring us to the Muller (2014) paper. We will include this study in the revised manuscript. Notably, as the reviewer correctly mentioned, the traveling wave velocity reported in the monkey visual system in their study is close to our findings in the human hippocampus. Specifically, using our approximate expression for velocity in the frequency range examined by Muller et al. (5–20 Hz): $velocity\sim0.02*freq$, we estimate a range of 0.1–0.4 m/s. In comparison, their reported velocity range was 0.25–1.35 m/s, indicating faster flow in the visual cortex of the monkey.

In Muller et al., traveling waves were measured using a phase-based method that, in many respects, is similar to our approach. They utilized the Hilbert transform to convert the band-pass filtered time series into amplitude and phase, while we used the Wavelet transform. The Hilbert transform is commonly used for extracting instantaneous amplitude, phase, and frequency and is well-suited for narrowband signals. In contrast, Wavelet transformation decomposes a signal into time-frequency components, making it suitable for analyzing both time-localized and multi-frequency signals.

In their study, the signals were band-pass filtered in the range of 5–20 Hz, which made the Hilbert transform an appropriate and straightforward choice. In our case, however, we specifically investigated differences across multiple frequencies, for which the Wavelet transform was more appropriate due to its ability to analyze time-frequency variations.

Another significant difference between Muller’s approach and ours lies in the scope of the analysis. Muller et al. employed a bivariate (pairwise) method applied to two-dimensional data, while our method is multivariate, and was applied to one-dimensional data. We argue that to obtain accurate results, a multivariate analysis is essential, especially when analyzing pathways consisting of several nodes. Using pathways ensures that phase differences and distances along effectively unidirectional pathways are correctly calculated. In contrast, bivariate analysis could result in a mixture of coherences that biases the phase and distance estimations, leading to incorrect velocity calculations. This point is extensively discussing in the paper to explain the difference in estimated velocities between bivariate and multivariate analyses.

However, when using two- (or three-) dimensional data, it is possible to correctly identify traveling waves, and thus to calculate the phase and distances correctly. This is since the observation of 2D traveling waves inherently accounts for the coherence mixture. Thus, the analysis in Muller et al.'s paper is valid and its estimated velocity is similar to ours.

Furthermore, a more recent study by Mohan et al. (Nat Hum Behav, 2024), cited in our manuscript, used a similar 2D-like approach with EEG data from the human cortex. Their velocity estimates align closely with ours, reinforcing the reliability of our methodology.

3. Second, regarding the physiological role of traveling waves, the same authors as above (Chemla et al. J Neurosci 2020) showed that creating "collisions" between traveling waves in monkey V1 reveals a suppressive component. This suppression was found to help discriminating ambiguous stimuli, so here the waves seems to augment visual acuity. I think this possible role for traveling waves should be mentioned at least. The question is whether you can infer such suppressive (or amplifying) interactions between multiple waves using your method, can you please clarify?

We agree that it is appropriate to include in the introduction the role of traveling waves in addressing the "correspondence problem." This addition will be made—thank you for the suggestion.

Regarding the use of multivariate pathway analysis to test the “correspondence problem”, this requires further consideration and experimental validation. As noted earlier, the strengths and limitations of our approach lie in its ability to characterize nonlinear, continuous interactions that define pathways of information flow, while acknowledging that these pathways are inherently one-dimensional.

We will seek data where two stimuli (either simultaneously or sequentially) are expected to initiate synchronous activity at distinct locations, propagate along pathways, and converge at certain points. These meeting points can be conceptualized as hubs that initiate new pathways or as terminus points for the pathways.

Our initial approach to investigate this will involve a straightforward occurrence strategy: calculating the probability of different locations (ROIs) based on their positions within the pathways (e.g., first, second, third, or fourth) and calculating their coherence strength.
